# A novel approach for pathogen reduction in wastewater treatment

**DOI:** 10.1186/2052-336X-11-12

**Published:** 2013-06-28

**Authors:** Dhevagi Periasamy, Anusuya Sundaram

**Affiliations:** 1 Department of Environmental Sciences, Directorate of Natural Resources Management, Tamil Nadu Agricultural University, 641003, Tamil Nadu, India

**Keywords:** Hospital wastewater, Pathogen reduction, Specific phage, Wastewater treatment

## Abstract

Many sewage waste treatment systems are aiming for complete pathogen removal which necessitates search for novel approaches that does not harm the environment. One such novel approach is exploring the possibilities of bacteriophages for pathogen removal. Hospital wastewater was collected from different locations of Tamil Nadu and used for the study. The total heterotroph and total coliform population ranged from 1.6 × 10^5^ to 8.3 × 10^6^ per mL and from 1.2 × 10^3^ to 1.6 × 10^3^/ 100 mL of sample respectively. Higher frequency of antibiotic resistant *E. coli, Pseudomonas sp. Streptococcus sp* and *Bacillus spp* were observed in all the places, which clearly indicated the extent of pollution. All the samples had specific phages against *E. coli and* none of the samples had phages against MTCC culture. *E. coli* specific phage was isolated and the population of phage required for effective killing of *E. coli* has been standardized as 3 × 10^4^ pfu / mL of lysate. The inoculation resulted in 100% removal of pathogen from sewage water within 14 hours of incubation.

## Introduction

From the early 1970 to about 1990s, wastewater treatment objectives were based primarily on aesthetic and environmental concerns. At present the earlier objectives of reduction and removal of BOD, suspended solids, and pathogenic microorganisms continued, but at higher levels. Several developed and developing countries embarked on programmes to reduce waterborne multidrug resistant bugs (MDR). Presently R-plasmid carrying bacteria are a major cause of hospital borne infections. Indiscriminate release of hospital wastewater in the sewage system paves way for the entry of Multidrug Resistant bacteria in the sewage.

The greatest fear was the transfer of resistance to pathogens like *S. typhi*, which came true in 1972 resulting in an epidemic of chloramphenicol - resistant *S. typhi* and in 1992 another epidemic with simultaneous resistance for chloramphenicol, co-trimoxazole and ampicillin [1]. The main cause for the emerging MDR in sewage is heavy use of antibiotics and indiscriminate release of hospital wastewater into public sewage [2,3].

The dangers of infectious hospital waste received a great deal of attention in the last decade and main emphasis was given to hospital solid waste; but liquid waste released into the sewage has not received much attention. Mandatory monitoring of water quality and suitable disinfection measures should be done on a regular basis and not as an adhoc practice.

Very recently the ability of phages to control bacterial population has extended from the fields of medicine, agriculture, aquaculture, food industry into wastewater treatment also. Commercial production of a phage to kill *E. coli* O157: H7 in manure and to remove pathogen from carcasses and food preparation areas is already underway [4]. So there is the potential application of phages in wastewater treatment system to improve effluent quality and sludge disposal into the environment. Hence the following study has been initiated to explore the possibilities of utilizing the specific phages as biocontrol agents against the potential pathogens in hospital wastewater.

## Materials and methods

### Characterization of wastewater

Hospital wastewater samples were collected from eight locations of Tamil Nadu and subjected to characterization to see the magnitude of pollution. The samples were collected from the following places viz., Kovai Medical Centre & Hospital, Coimbatore; Government Hospital, Coimbatore; Government Hospital, Erode; Government Hospital, Salem; Government Hospital, Theni; Government Hospital, Trichy; Government Hospital, Chennai and Christian Medical College, Vellore. Three wastewater samples from each of the eight places were collected from outer most ends before the drainage flows to the municipal sewage. Before collecting the sample precautions were taken to avoid the infection. With the help of sanitary workers, working in different places, the samples were collected in presterilsed containers and transported to the laboratory for characterization as per the standard method [5].

### Bacteriological analysis of hospital wastewater

All the samples were subjected to viable count studies by spreading 100 μl of 10^–1^ to 10 ^– 12^ dilution prepared in sterile saline over the nutrient agar plate. The plates were incubated overnight at 37°C and plates showing 50 to 200 colonies were used for expressing the total viable bacterial count. The bacteriological analysis like the number of bacterial colonies, number of total coliform, and faecal coliform were measured by standard plate count (SPC), most probable number (MPN) and faecal coliform count (FCC) respectively. The samples were also plated in specific media to isolate the potentially dreadful pathogens [6] and subjected to further characterization to identify the organisms as per the standard procedures [7,8].

The antibiotic resistance of the strains was tested using disk diffusion test [9]. For the estimation of the MDR bacteria, 100 μl diluted samples were spread over MacConkey agar plates supplemented with 30 μg/mL of chloramphenicol and 20 μg/mL of gentamycin.

### Isolation of specific bacteriophages against the target pathogens

Enrichment was done to increase the number of phage virions in hospital waste water using phage decca double strength broth using *E. coli* as host cells. Phages in the filtrate were determined by seeding - agar overlay method [10]. When confluent lysis has occurred, 5 mL of SM buffer was added to the plate and gently scrape the soft agarose into sterile centrifuge tube. Tubes were spun at 4000 rpm for 10 min at 4°C, and the supernatant was recovered, to that one drop of chloroform was added to lyse the remaining cells. Thus prepared bacteriophages were maintained as stock.

### Characterization of the identified bacteriophages

Bacteriophages were titrated with their respective dilutions to know the number of plaques formed for their respective host and results were observed. Multiplicity of infection test is essential for fixing the time of treatment, dose of the phage dilutions to be used for wastewater disinfection [10]. Bacteriophages are highly specific and to check the specificity of the phages, few cultures were obtained from MTCC, Chandigarh and tested against the phages isolated from sewage (Table 1).

**Table 1:** Isolation of specific phages for MTCC cultures

**MTCC code**	**Name of the organism**
86	*Serratia marcescens*
98	*S. typhimurium*
3917	*Salmonella typhi*
740	*Staphylococcus aureus*
1302	*Escherichia coli K-12*
1303	*Escherichia coli B*
1588	*Eschericha coli CSh 57*
1650	*Escherichia coli KL 16*
1652	*Eschrichia coli DH5 α*
1748	*P. fluorescens*
310	*S. cerevisiae*
7299	*Proteus vulgaris*
7664	*E. aerogenes*

### Utilization of the bacteriophages as biocontrol agents against potential pathogen in sewage water

Enumerated bacteriophages were tested for the biocontrol efficacy in controlling the target pathogens. The test organism selected for the study was *E. coli*. The target pathogens were inoculated separately as well with specific bacteriophages and time course study was done to know about the survival rate of pathogens.

The selected organisms were inoculated into Lactose broth and sewage water. Since hospital wastewater is going to end up with sewage system, sewage water collected from Ukkadam in Coimbatore was used for the study. Sewage water is sterilized before introducing the target organism which helps to know the influence of other native organisms during the phage treatment. The following are the treatments used for the study.

T1 - ( Control) LB with *E. coli*

T2 - Sewage water with *E. coli*

T3 - Sterile sewage water with *E. coli*

T4 - T1 and *E. coli specific* bacteriophages

T5 - T2 and *E. coli specific* bacteriophages

T6 - T3 and *E.Coli specific* bacteriophages

Sewage water was collected and filtered, then sterilized in an autoclave to free the native organism. Sewage sample (100 mL) was taken in Din thread screw bottles, sterilsed and inoculated with *E. coli* at @ 10^4^ / ml. After inoculation, cell count of the inoculated pathogen was assessed to test the phage efficacy. This helps to fix the phage concentration during the scale up process. If the colony forming units exceeded 300; it is denoted as uncountable numbers (UC). Serial dilutions were carried up to 10 dilutions. From the serially diluted samples, 0.1 mL of pathogenic cultures were added to sterile plates containing LB (with sewage extract and without sewage extract) and incubated at 37°C for 24 hours. The pathogen survival was studied at every 1 hour interval and upto 14 hours the survival was assessed.

### Developing an eco-friendly bioconsortium for augmenting the pathogen in sewage water

The *E. coli* and *Salmonella typhi* organisms were inoculated into sewage water. Sewage water collected from Ukkadam was used for the study. The following are the treatments

T1 - Sewage water inoculated with *E. coli* and *E. coli s*pecific bacteriophages

T2 - Sewage water inoculated with *S. typhi and S. typhi* specific bacteriophages

T3 - Sewage water inoculated with *E. coli* and *Salmonella typhi* specific bacteriophages

T4 - Control

After filtration 100 ml of sewage sample was taken in Din thread screw bottles and sterilized. After cooling it was inoculated with *E. coli* at @ 10^4^ / ml and *Salmonella typhi at* @ 10^3^ / mL. After inoculation the pathogen survival was assessed at 14 hours.

## Results and discussion

Samples were collected from various Hospital wastewater and target microorganisms were isolated and identified.

### Characterization of wastewater

Physico chemical characteristics of the collected samples were analyzed and the results are presented in Table 2. All the samples collected had acceptable level of pH, but high COD level and very low dissolved oxygen ranging from 2.14 to 4.82 mg/L was recorded.

**Table 2:** Physicochemical characterization of hospital wastewater

**S.No**	**Name of the hospital**	**pH**	**TSS (mg/L)**	**DO (mg/L)**	**BOD (mg/L)**	**COD (mg/L)**
1	Kovai medical centre & hospital, Coimbatore	6.89	147.0	2.40	56.47	658.74
2	Government hospital, Coimbatore	6.48	138.2	4.82	124.4	724.82
3	Government hospital, Erode	7.40	85.6	2.42	132.8	623.47
4	Government hospital, Salem	6.42	148.4	3.68	146.4	542.21
5	Government hospital, Theni	8.12	65.8	4.22	84.6	548.32
6	Government hospital, Trichy	7.98	104.9	3.87	92.8	627.81
7	Government hospital, Chennai	8.25	198.8	2.14	248.3	849.92
8	CMC hospital, Vellore	8.45	124.3	4.62	148.9	728.24

Physiochemical parameters studied revealed that the hospital wastewater though show some parameters within the WHO standards, other parameter, whose values are higher than the WHO acceptable limits. Therefore, contamination of the receiving environment (water, soil and air) due to the discharged hospital wastewater, which could probably be hazardous to human health. In our country 70% of the water is seriously polluted and 75% of illness and 80% of the child mortality is attributed to water pollution [11,12]. The improper management of water systems may cause serious problems in availability and quality of water [13]. The healthy nature of underground water has also been altered [14-16]. Aluyi *et al.*[17] investigated the bacteriological and physiochemical qualities of hospital wastewater and observed the same results as that of the present study.

### Bacteriological analysis of hospital wastewater

The main objective behind the bacteriological anlysis is to determine the microbial pollution, which is a paramount in assessing the associated health risks. The bacteriological analysis like the number of bacterial colonies, number of total coliform, and faecal coliform were measured (Table 3). The total heterotrophic bacterial counts, ranged from 1.9 × 10^7^ to 8.3 × 10^12^ cfu/mL and total coliform counts ranged from 1.2 × 10^3^ to 1.6 × 10^3^ MPN/100mL.

**Table 3:** Bacteriological analysis of hospital wastewater

**S.No**	**Name of the hospital**	**Total heterotrophic bacterial count (SPC/TPC)**	**Total coliform count(TCC)**	**Fecal coliform count (FCC)**
1	Kovai medical centre and hospital, Coimbatore	6.7 × 10^5^	1.6 × 10^3^	16.06 × 10^1^
2	Government hospital, Coimbatore	8.3 × 10^6^	0.92 × 10^3^	24.28 × 10^1^
3	Government hospital , Erode	2.6 × 10^5^	1.6 × 10^3^	2.51 × 10^1^
4	Government hospital, Salem	8.8 × 10^4^	0.92 × 10^3^	2. 56 × 10^1^
5	Government hospital, Theni	1.9 × 10^3^	1.2 × 10^3^	1.1 × 10^2^
6	Government hospital , Trichy	3.6 × 10^5^	1.6 × 10^3^	3.2 × 10^2^
7	Government hospital, Chennai	8.6 × 10^5^	> 2.4 × 10^3^	26.06 × 10^1^
8	CMC hospital, Vellore	1.6 × 10^5^	1.6 × 10^3^	1.8 × 10^1^

Value represents mean of three replications.

For presence of pathogenic bacteria, the coliform group of bacteria can be detected by testing the sample. The more number of faecal coliform indicated the presence of faecal material from warm blooded animals. All the water samples were contaminated with more number of faecal coliforms, which is in accordance with Rajurkar *et al.*[18]. The reason for the high number of faecal streptococci might be due to addition of human and warm blooded animal’s excretae [19]. According to WHO estimate about 80% of water pollution in developing country, like India is carried by domestic waste and about 95% of rural population living in India depends on ground water for domestic use [20].

**Figure 1:**
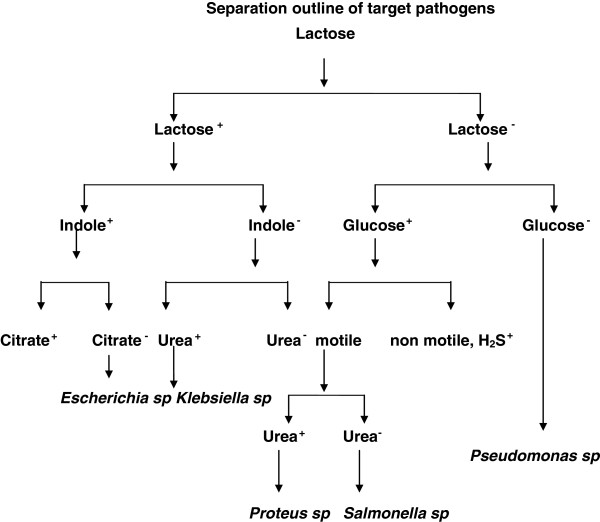
Separation outline of target pathogens.

Untreated wastewater contains numerous disease causing microorganisms and toxic compounds that dwell in the human intestinal tract may contaminate the land or water body where hospital waste is disposed. Qualitative analyses were used to determine the sanitary condition of the water. The samples were also plated in specific media to isolate the potentially dreadful pathogens using the following separation outline (Figure 1).

The bacterial isolates were isolated and characterized *Klebsiella, Pseudomonas, Escherichia, Serratia, Staphylococcus, Streptococcus, Proteus* and *Bacillus*. *Klebsiella, Pseudomonas* and *Serratia* were the most frequently distributed isolates in the hospital wastewater (Table 4).

**Table 4:** Isolation of microorganisms using specific media

**S.No**	**Samples**	**Colony forming units X 10**^**2 **^**/mL of sample**										
		***E. coli***	***S. sp***	***Ps. sp***	***K. sp***	***Staph. sp***	***Strep. sp***	***Proteus sp***	***Bacillus *spp**	**Aspergillus x 10**^**1**^	***Azotobacter *x 10**^**1**^	***Yeast *x 10**^**1**^
1	KMCH Cbe- 3	84	32	-	1	-	-	-	28	24	-	4
2	GH, Cbe-3	102	14	4	-	-	-	-	48	4	-	-
3	GH, Erode	12	-	-	-	-	-	-	36	24	42	8
4	GH, Salem	44	4	2	2	-	1	1	107	-	-	4
5	GH, Theni	30	ND	ND	ND	-	-	-	28	-	-	-
6	GH, Trichy	32	7	ND	ND	-	-	-	94	8	-	-
7	GH, Chennai	160	8	2	1	4	2	-	92	4	-	12
8	CMCH Vellore	174	2	-	ND	-	-	4	49	2	4	-

The MDR problem encountered in hospitals is mainly due to Gram-negative bacteria. Hence for the estimation of the MDR bacteria, 100 μl diluted samples were spread over MacConkey agar plates supplemented with 30 μg/mL of chloramphenicol and 20 μg/mL of gentamicin. Chloramphenicol and gentamicin were selected because they represent two of the commonly used antibiotics over the last thirty years and also have greater *invitro* stability. Differentiation as lactose fermenter and non-lactose fermenter could be made on Mackonkey agar for MDR isolates. A minimum of three colonies with similar morphology were selected individually and subjected to identification by standard biochemical methods and also subjected to drug susceptibility by the disk diffusion technique [21].

Simultaneous resistance to Ciproflaxin, Tetracycline, Streptomycin, Kanamycin, Ampicillin, Erythromycin, Penicillin, Cephalosporin and Rifampicin formed the common MDR pattern (Table 5).

**Table 5:** Resistance patterns of MDR bacteria isolated from hospital wastewater

**S. No**	**Antibiotics**	**KMCH Cbe**	**GH, Cbe**	**GH, Erode**	**GH, Salem**	**GH, Theni**	**GH, Trichy**	**CMCH Vellore**	**GH, Chennai**
1	Ciproflaxin (10 mcg)	I	I	R	S	R	R	R	S
2	Tetracycline (30 mcg)	R	R	R	S	S	I	R	I
3	Streptomycin (10 mcg)	S	R	I	S	I	R	I	S
4	Kanamycin (10 mcg)	S	R	S	R	I	S	I	R
5	Ampicillin (10 mcg)	I	R	R	I	R	R	I	S
6	Erythromycin (15 mcg)	R	R	S	S	R	I	R	R
7	Penicillin (10 mcg)	I	R	S	R	R	S	S	R
8	Cephalosporin (30 mcg)	R	R	R	R	R	R	R	S
9	Rifampicin (5 mcg)	S	S	I	R	I	R	I	S

*R* Resistant, *S* Sensitive, *I* Intermediate resistant.

Some hospital wastewater samples especially, government hospital, Coimbatore showed very high percentage of MDR bacteria. The antimicrobial selective pressure through indiscriminate use of antibiotics has played a significant role in enriching the MDR R + strains in the hospital wastewater. A sizeable number of hospital strains have become resistant simultaneously to most of the available antibiotics [22,23]. Low loads of liquid waste generated due to scarcity of water may also be one of the reason for increased population. The worst fear apprehended is the transfer of such resistance to bacterial pathogens causing infections in the community. The present observations suggest that hospital effluents can be a potential health hazard by adding MDR bacteria to a city sewage pool.

### Isolation of specific bacteriophages for target pathogens

Host specificity is central to selection of suitable phages for wastewater treatment applications [24]. Success would depend on accurate identification of problem, effective isolation and unbiased enrichment of phage and ability of phage to penetrate flocs and remain infective in *insitu* condition. The target bacteria used is *E. coli* (Table 6) and phages specific to *E. coli* was selected by agar overlay method.

**Table 6:** **Morphological and biochemical characteristics of *E. coli***

**S. No**	**Tests performed**	**Results**
1	Shape	Rods
2	Gram staining	Gram negative
3	Motility	Motile
4	Gelatin utilization test	Negative
5	Citrate utilization test	Positive
6	Methyl red	Negative
7	Voges proskeur test	Positive
8	Acid from glucose	Positive
9	Gas from glucose	Negative
10	Triple sugar Iron test	Acid was produced
11	Urease test	Positive
12	Indole production	Negative

Values in parenthesis indicate the drug concentration in mcg/disc.

To determine the plaque formation, double layer agar plates were prepared as it is essential for the differentiation between formation of clear plaques and turbid plaques. Many bacteriophages require divalent cations such as Mg^++^ and Ca^++^ for attachment to bacterial host cells. Hence it is essential to grow in bacterial growth medium with 10 mM MgSo_4_ and 0.2% Maltose. During the transport of these ions and carbons into the cells through Porin, phage particles also can enter the cells. Magnesium and Maltose facilitates the entry of phage particles into the cell [25].

Plaque formation was observed due to the inhibition of growth and lyses of the phage infected cells in the bacterial Lawn. Based on the ability of bacteriophages to lyse bacterial cells, phages were grouped into host sensitive/ resistant phages. If specific phages infected and lysed the host cells, a spontaneous clear plaque variant was formed. The clear plaque variant was purified several times and on further infection of the host cells [26].

### Characterization of the identified bacteriophages

#### Titration of Bacteriophages

Bacteriophages were titrated to know the number of plaques formed for the respective host (*E. coli*). The phages were serially diluted up to 10^6^ in LB broth and from the serially diluted phages, 0.1 mL was mixed with 0.2 mL of *E. Coli* in separate tubes up to 10^6^ dilutions. Meantime soft agar was sterilized and maintained at 42-45°C in a water bath. The soft agar was added to the consecutive dilutions and plated on solid LB agar. The plates were allowed to solidify. The solidified plates were incubated at 37°C. After incubation the plates were observed for plaque forming units and titration was tabulated (Table 7).

**Table 7:** **No. of plaque forming units per mL of the *E. coli *lysate**

**S. No**	**Dilution factor**	**pfu/ mL of sample**
1	10^-2^	TNC
2	10^-3^	175 × 10^5^
3	10^-4^	116 × 10^6^
4	10^-5^	83 × 10^7^
5	10^-6^	74 × 10^8^

Values represent mean of three replications. *TNC* Too Numerous to Count

#### Isolation of specific phages for MTCC cultures

Bacteriophages are highly specific and to check the specificity of the phages, cultures were obtained from MTCC (Table 1), Chandigarh and tested against the phages isolated from sewage. The results indicated that none of the samples had bacteriophages against MTCC cultures and shows the specificity [27,28].

### Utilization of bacteriophages as biocontrol agents against potential pathogen in sewage water

Biological hazard in water resources in the form of pathogenic organisms are responsible for major outbreak in most of the developing countries. In this situation, every effort leading to reduction in sewage pollution and pathogenic microbes has to be promoted and implemented. This will not only safeguard the interest of the people but also help to maintain healthy and sustainable environment. Entry of antibiotic resistant pathogens into the sewage is inevitable as survival is the key for existence. Development of multidrug resistant bacteria and exit of many antibiotic companies necessitates to search for novel approaches to tackle the multidrug resistant bacteria. Phage therapy is an alternate to overcome these menacing organisms.

It is essential for the success of any phage therapy; suitable phage should be isolated, enriched to produce sufficient numbers for the application. The number of bacteriophages to be inoculated should be 3 to 10 times greater than bacteria [29]. Payne and Jansen [30] observed that insufficient host cell concentration may also contribute for phage decline. Phage enrichment normally involves the inoculation of mixed environmental samples and growth media with single host strain. Repeated phage purification using just one host strain may increase the specificity for that strain [31-33].

In order to fix the dose of host cells *E. coli* broth was diluted to assess the cell count. In case of *E. coli* upto 10^-4^ dilutions there are uncountable numbers. Countable numbers were observed only in 10^-8^ and 10^-9^ dilutions. Upto 10^-3^ dilutions, colonies formed were too numerous to count (TNC). Sewage water inoculated with *E .coli* (T2) and sterile sewage (T3) also had less population, which shows the native environmental influence as well as limited availability of nutrients. After phage inoculation not much change was observed up to 2 hr of incubation (Tables 8 and 9).

**Table 8:** **Cell count of *E. coli *(cfu/mL) in sewage water at 1 hour**

**1 hour**						
	10^-4^	10^-5^	10^-6^	10^-7^	10^-8^	10^-9^
.T1	UC	249	86	94	48	12
T2	198	98	76	43	6	-
T3	126	48	16	-	-	-
T4	UC	194	108	82	54	36
T5	186	83	80	28	-	-
T6	138	56	42	12	-	-

Values represent mean of 3 replications.

**Table 9:** **Cell count of *E. coli *(cfu/mL) in sewage water at 2 hours**

**2 hours**						
	10^-4^	10^-5^	10^-6^	10^-7^	10^-8^	10^-9^
T1	UC	UC	220	195	142	85
T2	260	147	138	56	12	-
T3	134	86	42	4	-	-
T4	UC	186	110	68	47	24
T5	UC	198	102	94	82	41
T6	UC	94	50	28	6	-

Values represent mean of 3 replications.

There was steady increase in the host population in T1, T2 and T3 treatments after 4 hours of inoculation. In case of treatments inoculated with phages, the host population was maintained without increase in the population (Table 10). After 6 hours, in uninoculated treatments, there was steady increase in the population, whereas in phage treated samples slight reduction in host population was observed (Table 11). The effect was more pronounced in treatment 4 and 6. This shows the specificity and T5 has non specific *E. coli* also. In treatments T1 – T3, there was steady increase in the host population, whereas phage inoculated treatment (T4 – T6) drastic reduction in population was observed after 8 and 10 hours of inoculation (Tables 12 and 13). After 12 hours of inoculation itself, the reduction was so high (Table 14) and after 14 hours the host population is completely vanished (Table 15).

**Table 10:** **Cell count of *E. coli *(cfu/mL) in sewage water at 4 hours**

**4 hours**						
	*10*^*-4*^	*10*^*-5*^	*10*^*-6*^	*10*^*-7*^	*10*^*-8*^	*10*^*-9*^
T1	*UC*	*UC*	*UC*	*248*	*147*	*94*
T2	*UC*	*UC*	*240*	*124*	*44*	*-*
T3	*UC*	*108*	*64*	*26*	*14*	*-*
T4	*UC*	*98*	*78*	*43*	*24*	*-*
T5	*168*	*94*	*68*	*24*	*-*	*-*
T6	*120*	*47*	*13*	*-*	*-*	*-*

Values represent mean of 3 replications.

**Table 11:** **Cell count of *E. coli *(cfu/mL) in sewage water at 6 hours**

**6 hours**						
	10^-4^	10^-5^	10^-6^	10^-7^	10^-8^	10^-9^
T1	UC	UC	UC	UC	198	142
T2	UC	UC	UC	268	194	120
T3	UC	248	164	124	64	14
T4	140	88	74	46	20	-
T5	148	96	64	19	-	-
T6	118	38	14	-	-	-

Values represent mean of 3 replications.

**Table 12:** **Cell count of *E. coli *(cfu/mL) in sewage water at 8 hours**

**8 hours**						
	10^-4^	10^-5^	10^-6^	10^-7^	10^-8^	10^-9^
T1	UC	UC	UC	UC	274	184
T2	UC	UC	UC	UC	248	169
T3	UC	UC	268	194	112	86
T4	28	18	-	-	-	-
T5	124	48	12	-	-	-
T6	64	28	4	-	-	-

Values represent mean of 3 replications.

**Table 13:** **Cell count of *E. coli *(cfu/mL) in sewage water at 10 hours**

	**10 hours**					
	10^-1^	10^-2^	10^-3^	10^-4^	10^-5^	10^-6^
T1	UC	UC	UC	UC	UC	UC
T2	UC	UC	UC	UC	UC	UC
T3	UC	UC	UC	UC	UC	268
T4	247	196	68	12	3	-
T5	UC	UC	268	88	32	4
T6	UC	248	184	35	12	-

Values represent mean of 3 replications.

**Table 14:** **Cell count of *E. coli *(cfu/mL) in sewage water at 12 hours**

**12 hours**						
	10 ^-1^	10 ^-2^	10 ^-3^	10 ^-4^	10 ^-5^	10 ^-6^
T1	UC	UC	UC	UC	UC	UC
T2	UC	UC	UC	UC	UC	UC
T3	UC	UC	UC	UC	UC	UC
T4	48	23	4	-	-	-
T5	112	64	18	4	-	-
T6	116	94	28	12	-	-

Values represent mean of 3 replications.

**Table 15:** **Cell count of *E. coli *(cfu/mL) in sewage water at 14 hours**

**14 hours**						
	10 ^-1^	10^-2^	10^-3^	10^-4^	10^-5^	10^-6^
T1	UC	UC	UC	UC	UC	UC
T2	UC	UC	UC	UC	UC	UC
T3	UC	UC	UC	UC	UC	UC
T4	3	-	-	-	-	-
T5	16	-	-	-	-	-
T6	8	-	-	-	-	-

Values represent mean of 3 replications.

As the time of incubation increases the host population was also increased in lactose and sewage water samples, whereas in other treatments not much increase was observed. The target population increased, but in phage treated samples not that much increase was observed and this may be due to adsorption of phage particles and it may change the metabolic rate of the target pathogens. In uninoculated treatments there was steady increase in the target population. This shows that phage reduced the target population. Based on the single step growth experiment, with in 7 to 8 hours, the phage population reached the maximum level. So the incubation time in this experiment was maintained up to 14 hours.

### Developing an eco-friendly bioconsortium for augmenting the pathogen in sewage wastewater treatment

Phage mediated bacterial mortality has the capacity to influence treatment performance by controlling the abundance of key functional groups. As a preliminary study the developed bacteriophage preparations were tested in sample collected at Coimbatore Corporation Ukkadam sewage treatment plant. The characteristics of the wastewater from the outlet of sewage treatment plant are given in Table 16.

**Table 16:** Quality of water treated at Ukkadam STP

**Parameter**	**Raw sewage quality**	**Treated sewage quality**
BOD	250 ppm	< 10 ppm
COD	580 ppm	< 100 ppm
Total nitrogen	15 ppm	< 10 ppm
Total phosphorus	5 ppm	< 2 ppm
Fecal Coliform	10^6^ nos / 100 mL	< 200 nos / 100 mL
pH	7.5	7.9

The lysate of *E. coli* and *Salmonella typhi* phages were mixed and used for the treatment. After 14 hours of incubation, there was no *E. coli* and *Salmonella typhi* population in the wastewater (Table 17). Hantula *et al.*[34] found that approximately 10% of phages isolated from activated sludge were polyvalent in nature.

**Table 17:** Effect of phage consortium on pathogens

**S.no.**	**Treatment details**	**Initial population**		**After treatment (14 hours)**	
		***E.coli***	***Salmonella typhi***	***E.coli***	***Salmonella typhi***
T1	Sewage water inoculated with *E. coli* and *E. coli s*pecific bacteriophages	2486	35	nil	22
T2	Sewage water inoculated with *Salmonella sp* and *Salmonella typhi* specific bacteriophages	2478	78	24	nil
T3	Sewage water inoculated with *E. coli* and *Salmonella typhi* specific bacteriophages	2469	65	Nil	nil
T4	Control	2587	89	85	102

Multiple host range isolation technique may be more effective at isolating polyvalent phages by avoiding the selection bias of single host methods [35-38]. Tanji *et al.*[39] also reported that viral decay and loss of infectivity may reduce the efficacy of phage treatment of wastewater. Reduction in phage population may occur due to adsorption of phage particles to sludge flocs. (Eg). 97% of coliphage may be associated with suspended particles which are transferred to sludge during settlement. Poor penetration in the sludge flocs may reduce the efficacy of phage treatment. Kim and Unno [40] showed Ingestion of viral particles by bacteria, protozoa and metazoan may contribute to phage loss should be addressed. In addition, radiation also reduces the numbers. So the host and phage ration should be maintained for the success of the treatment.

## Conclusion

Based on our data and thorough scanning of previous studies, It was observed that hospital wastes have negative influence on the microbiological and physiochemical parameters on the environment, suggests that the activities of hospital wastes in the environment is a major health and environmental threat. Even though all unit operations (physical, chemical and biological) were carried out in sewage treatment, chlorination is normally used to disinfect the treated sewage, but this may not kill all the pathogens.

This study highlights the potential to develop phage treatments for generalized control of bacterial populations and the role of non host cells in determining the success of phage treatment in wastewater treatment. Pathogen specific phage isolated from sewage had the potential to eliminate the dreadful pathogens. Thus indicating that phage based biocontrol could be a viable method of controlling pathogens in sewage water. Despite some of the potential hindrances to the phage treatment, the current awareness regarding phages indicates that phage application to wastewater treatment deserves attention.

## Competing interest

I would also like to share the following information with Editor-in-Chief.

The concept of this project is that conventional disinfection process kills all organisms including those involved in degradation, nitrification etc, where as phages kills only the specific organism. Since bacteriophages are highly specific, ability to kill antibiotic resistant pathogens there is the potential application of phages in waste water treatment system to improve effluent quality and sludge disposal into the environment. More than eighty years of research on phage - human and phage - animal interaction, has shown no evidence for negative impact of application of specific phage to the human. In addition, the effectiveness of phage treatment increases exponentially until the host is eliminated. Phages are omnipresent and have no adverse effect on human and animals.

The technology developed is highly useful to the City corporations and Panchayats which use common sewage treatment system. The Ukkadam sewage treatment plant at Coimbatore corporation has been supplied with phage for controlling the *Salmonella typhi* pathogen. Based on the success of the treatment process it will be extended for adoption to other Corporations and municipalities.

Despite some of the potential hindrances to the phage treatment, the current awareness regarding phages indicates that phage application to wastewater treatment deserves attention. Hence this is the right time to publish this paper to create wide awareness.

## Authors’ contribution

Dr. PD: Principal investigator, Proposed, and presented the project before the ministry for financial support, carried out most of the work. Ms. SA: Worked as Senior Research Fellow for one year. She was involved in applying the phages in the Ukkadam sewage treatment. Both authors read and approved the final manuscript.
